# Supplementary material for: DNA dependent protein kinase (DNA-PK) enhances HIV transcription by promoting RNA polymerase II activity and recruitment of transcription machinery at HIV LTR
Source: Oncotarget. 2020 Feb 18;11(7):699–726. doi: 10.18632/oncotarget.27487 (PMC7041937; doi:10.18632/oncotarget.27487)
Supplement: Supplementary file 1 [file oncotarget-11-699-s001.pdf]

DNA dependent protein kinase (DNA-PK) enhances HIV transcription by promoting RNA polymerase II activity and recruitment of transcription machinery at HIV LTR

SUPPLEMENTARY MATERIALS

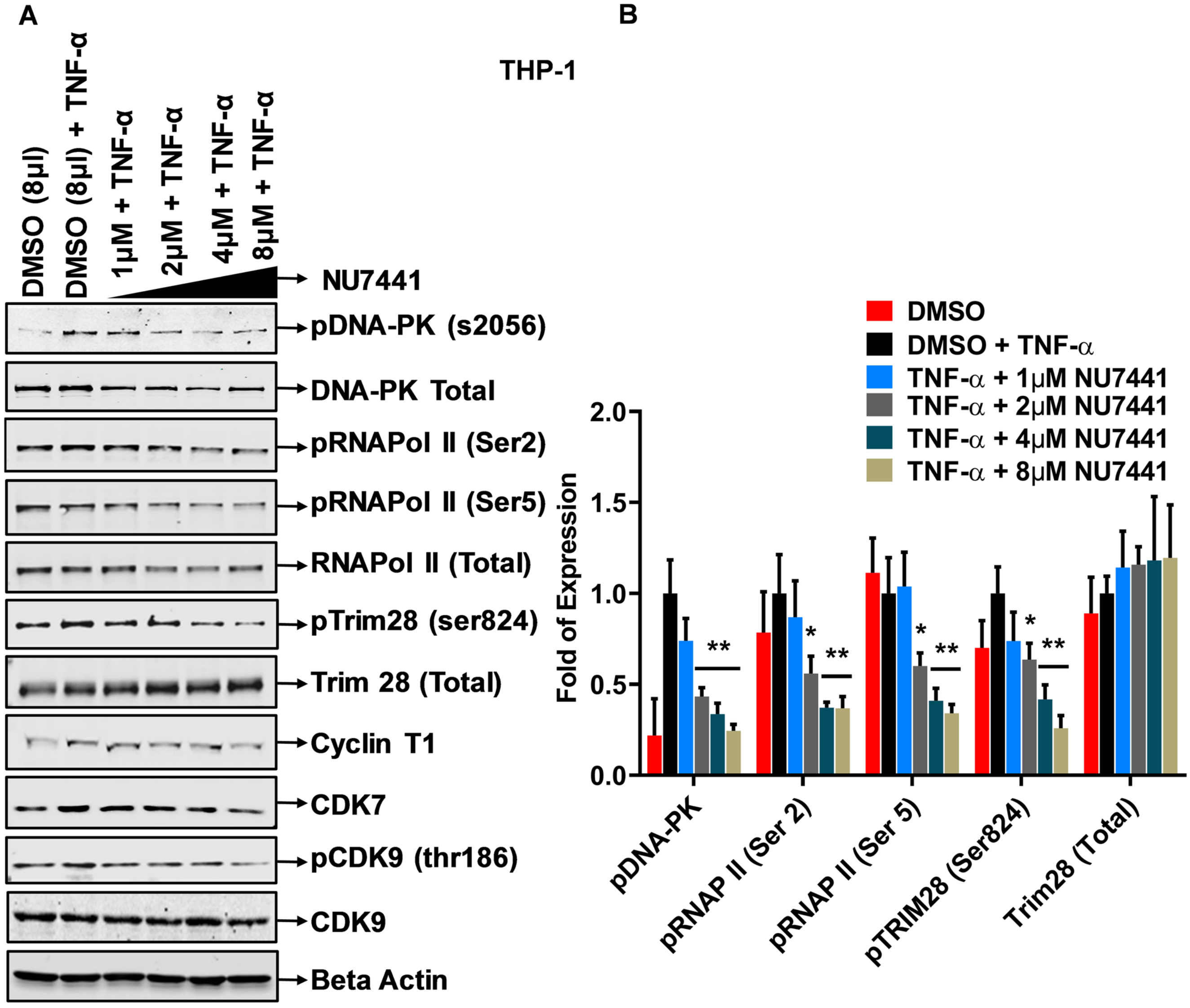

Supplementary Figure 1: DNA-PK inhibitors repress HIV transcription by restricting RNAP II CTD phosphorylation in THP-1 cells. (**A**) THP-1 cells harboring pHR'-PNL-wildTat-d2GFP provirus were treated with low concentrations of the DNA-PK inhibitor NU7441 (1–8 μM) and then activated with TNF-α (10 ng/ml) overnight. The nuclear lysates were analyzed by immunoblotting with the indicated antibodies, beta actin was used as loading control. (**B**) Densitometric analyses were performed on immunoblot bands using ImageJ software and represented graphically. Error bars represent the Mean ± SD of at least three separate experiments. The *p* value of statistical significance was set as $p < 0.05$ (*) or 0.01 (**) against the stimulated DMSO + TNF-α treated cells.

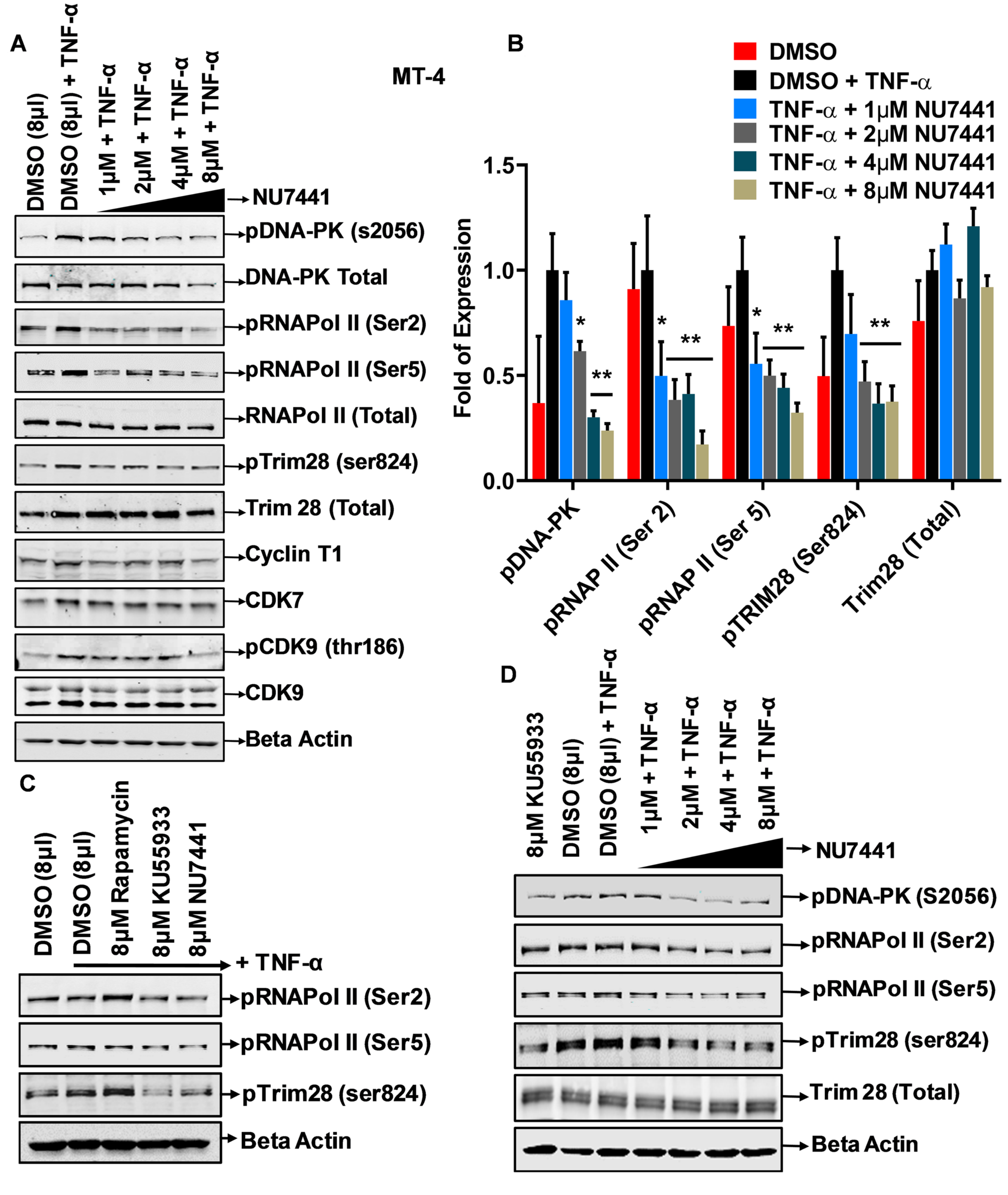

Supplementary Figure 2: DNA-PK inhibitors repress HIV transcription by restricting RNAP II CTD phosphorylation in MT-4 cells. (**A**) MT-4 cells harboring pHR'-PNL-wildTat-d2GFP provirus were treated with low concentrations of the DNA-PK inhibitor NU7441 (1–8 µM) and then activated with TNF-α (10 ng/ml) overnight. The nuclear lysates were analyzed by immunoblotting with the indicated antibodies and beta actin was used as loading control. (**B**) Densitometric analyses were performed on immunoblot bands using ImageJ software and represented graphically. The *p* values of statistical significance were set at either $p < 0.05$ (*) or 0.01 (**) against the DMSO + TNF-α treated cells. Error bars represent the Mean ± SD of at least three separate experiments. (**C**) Immunoblots using Rapamycin (mTOR inhibitor) and KU55933 (ATM inhibitor) as control (**D**) Immunoblot using KU55933 as one of the controls.

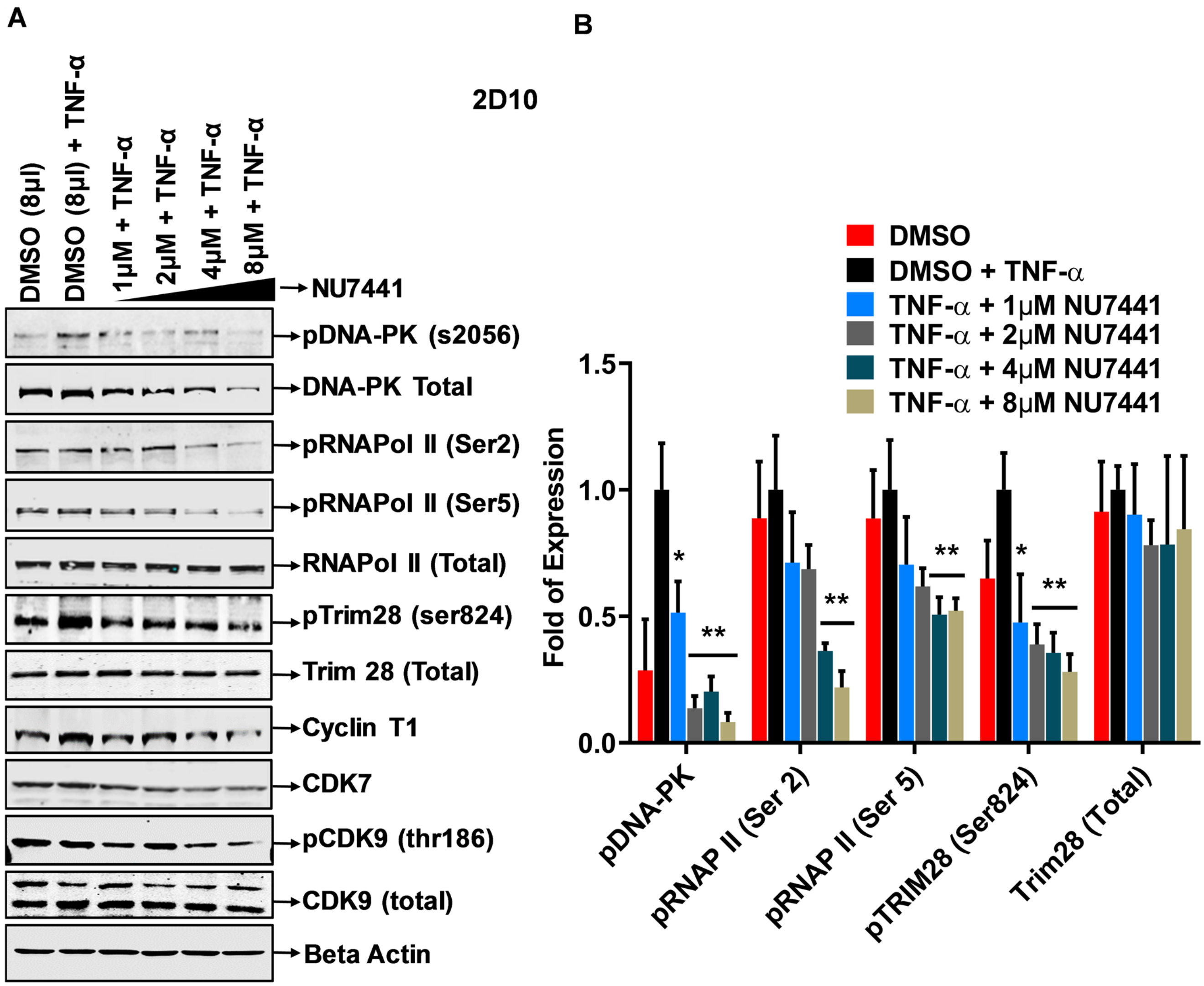

Supplementary Figure 3: DNA-PK inhibitors repress HIV transcription by restricting RNAP II CTD phosphorylation in 2D10 cells. (**A**) 2D10 cells carrying integrated latent pHR'-PNL-H13L-Tat-d2GFP provirus were treated with low concentrations of the DNA-PK inhibitor NU7441 (1–8 µM) and then activated with TNF-α (10 ng/ml) overnight. The nuclear lysates were analyzed by immunoblotting with the indicated antibodies and beta actin was used as loading control. (**B**) Densitometric analyses were performed on immunoblot bands using ImageJ software and represented graphically. The results were reproduced at least 3 times. Error bars represent the Mean ± SD of three separate experiments. The *p* values of statistical significance were set at either $p < 0.05$ (*) or 0.01 (**) against the DMSO + TNF-α treated cells.

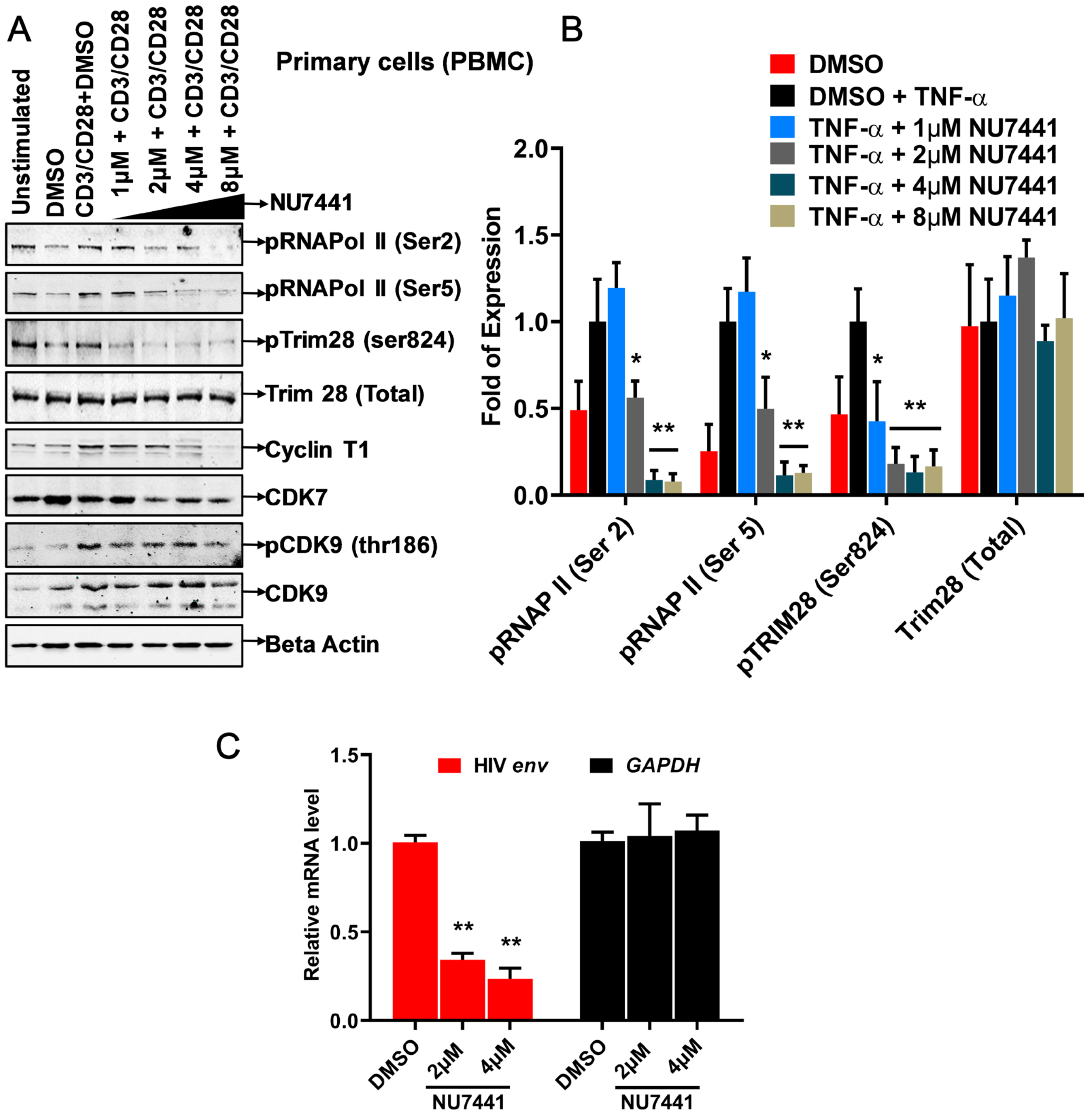

Supplementary Figure 4: DNA-PK inhibitors repress HIV transcription by restricting RNAP II CTD phosphorylation in primary cells. (**A**) pHR'-PNL-wildTat-d2GFP infected PBMCs were treated with low concentrations of the DNA-PK inhibitor NU7441 (1–8 μM) and then stimulated with α-CD3/CD28 antibodies overnight. The nuclear lysates were analyzed by immunoblotting with the indicated antibodies, beta actin was used as loading control. (**B**) Densitometric analyses were performed on immunoblot bands using ImageJ software, and represented graphically. Error bars represent the Mean ± SD of at least three separate experiments. The p value of statistical significance was set at either $p < 0.05$ (*) or 0.01 (**) against the DMSO + α-CD3/CD28 antibodies treated cells. (**C**) DNA-PK inhibitor inhibits HIV gene expression. MT-4 cells carrying integrated pHR'-PNL-wildTat-d2GFP provirus were treated with low concentrations of the DNA-PK inhibitor Nu7441 (starting from 2 and 4 μM) and then activated with TNF-α (10 ng/ml) for overnight. The cells were harvested, and mRNA were assessed. The relative expression levels of HIV mRNA in presence of DNA-PK inhibitor and control cells evaluated via RT-qPCR. The qPCR results represent the Mean ± SD of three independent assays. Statistical significance is set as $p < 0.01$ (**).
